# Oral Soft Tissue Biopsy in Patients Receiving Direct Oral Anticoagulants: A Prospective Cohort Study on Bleeding Outcomes

**DOI:** 10.1111/odi.70453

**Published:** 2026-08-04

**Authors:** Rossana Izzetti, Chiara Cinquini, Antonio Barone, Marco Nisi

**Affiliations:** ^1^ Department of Surgical, Medical and Molecular Pathology and Critical Care Medicine University of Pisa Pisa Italy

**Keywords:** anticoagulation, atrial fibrillation, direct oral anticoagulants, haemostasis, oral biopsy, oral surgery, perioperative bleeding, soft tissue biopsy, tranexamic acid

## Abstract

**Background:**

Direct oral anticoagulants (DOACs) are the first‐line therapy for stroke prevention in non‐valvular atrial fibrillation and venous thromboembolism. While evidence supports their continuation during routine dental procedures such as tooth extractions, data on oral soft tissue biopsies remain limited. The aim of this study was to evaluate bleeding rates in DOAC‐treated patients undergoing oral biopsy without drug discontinuation, compared to anticoagulant‐naive controls.

**Methods:**

One hundred patients were enrolled and divided into two groups of 50: DOAC group and control group. Both groups underwent standardised excisional soft tissue biopsy. Bleeding was assessed intraoperatively, perioperatively, and daily from postoperative day 1 (T1) to day 7 (T7) via a patient‐completed diary.

**Results:**

Intraoperative bleeding was significantly more frequent in the DOAC group (*p* < 0.05), as well as perioperative bleeding (*p* < 0.05). Postoperative bleeding was significantly higher in the DOAC group at T1 (22% vs. 6%, *p* < 0.05) and T2 (16% vs. 4%, *p* < 0.05), and moderate bleeding occurred exclusively in DOAC patients at T3 and T4 (4%, *p* < 0.05).

**Conclusions:**

Oral soft tissue biopsy in DOAC‐treated patients is associated with a higher risk of intra‐, peri‐ and postoperative bleeding compared to controls. However, bleeding severity remains mild to moderate and appears manageable in an outpatient setting.

**Trial Registration:**

The study protocol was registered in a clinical trials database (Clinicaltrials.gov) with registration number NCT07520890

## 1 Introduction

Direct oral anticoagulants (DOACs)—comprising the direct thrombin inhibitor dabigatran and the factor Xa inhibitors apixaban, rivaroxaban and edoxaban—have largely supplanted vitamin K antagonists (VKAs) as first‐line anticoagulant therapy for non‐valvular atrial fibrillation (NVAF) and venous thromboembolism (VTE) (Kearon et al. 2016; Hindricks et al. 2021). Their predictable pharmacokinetics, fixed dosing, absence of routine coagulation monitoring requirements and superior safety profile compared to warfarin have driven a rapid shift in prescribing patterns (Ballestri et al. 2023). In Italy alone, the 2022 Italian medicines agency (Agenzia Italiana del Farmaco, AIFA) report indicates approximately 1.8 million patients receiving oral anticoagulant therapy; notably, 80.4% of these patients are treated with DOACs.

The increasing prevalence of DOACs use inevitably means that oral and maxillofacial surgeons, as well as dental practitioners, will encounter a growing number of anticoagulated patients. Perioperative management of DOAC therapy requires balancing the thrombotic risk due to drug interruption and the haemorrhagic risk associated with its continuation (Spyropoulos and Douketis 2012). The 2021 European Heart Rhythm Association (EHRA) practical guide on the use of DOACs in patients with atrial fibrillation provides stratification of surgical procedures based on bleeding risk (Connolly et al. 2009; Patel et al. 2011; Granger et al. 2011) and classifies routine dental procedures—including simple tooth extractions, periodontal surgery, scaling and implant placement—among procedures with ‘minor bleeding risk’ (Steffel et al. 2021). Notably, oral soft tissue biopsies are not specifically addressed in these or in dental society guidelines, leaving clinicians without direct evidence to guide perioperative anticoagulation management (Steffel et al. 2021; Douketis et al. 2022). Importantly, oral lesions requiring histopathological diagnosis—including potentially malignant disorders, inflammatory conditions, vascular lesions and neoplasms—frequently occur in the elderly, a population that disproportionately receives anticoagulant therapy (Schnabel et al. 2015).

Current evidence on DOAC management in oral surgery relates almost exclusively to tooth extractions (Kumbargere Nagraj et al. 2018; Darwish 2023; Kim et al. 2024), although data on implant placement has emerged (Galletti et al. 2020; Zou and Hua 2023). Systematic reviews and meta‐analyses confirmed that patients on uninterrupted DOAC therapy undergoing simple extractions may experience mild, controllable bleeding that does not require hospitalisation, and that DOAC continuation is at least as safe as suspension for minor procedures (Galletti et al. 2020; Iwata et al. 2022; López‐Galindo and Grau‐Benítez 2023). However, soft tissue biopsies—which involve incision of vascular oral mucosa, excision of a lesion and suturing—carry a distinct bleeding profile, and evidence cannot be directly extrapolated from extraction studies.

Different classification systems have assigned oral biopsies to varying risk categories. The Scottish Dental Clinical Effectiveness Programme (SDCEP) (Woolcombe et al. 2022) classifies biopsies among procedures with high postoperative bleeding risk, while Nathwani et al. place them in the moderate‐risk category (Nathwani and Wanis 2017), and Serrano‐Sánchez and collaborators further stratify biopsy risk by lesion size (Serrano‐Sánchez et al. 2017). This heterogeneity draws the attention towards the absence of prospective data specifically addressing oral biopsies in DOAC‐treated patients.

The aim of the present prospective cohort study was to evaluate intraoperative, perioperative and postoperative bleeding outcomes in DOAC‐treated patients undergoing oral soft tissue biopsy without drug discontinuation, comparing them to a matched cohort of anticoagulant‐naive controls undergoing the same procedure.

## 2 Materials and Methods

### 2.1 Study Design and Ethics Considerations

This was a monocentric, prospective observational cohort study conducted at the Unit of Dentistry and Oral Surgery of the University Hospital of Pisa, Italy. The study protocol was approved by the institutional review board (Ethics committee North‐West Tuscany area, approval no. 17796/2020) and registered in a clinical trials database (NCT07520890). The study was conducted in accordance with the principles outlined in the Declaration of Helsinki. All participants provided written informed consent prior to enrolment.

### 2.2 Patient Selection

Consecutive patients referred for oral soft tissue biopsy between 2021 and 2025 were enrolled. Patients were allocated to the two study groups based on anticoagulant therapy status:

– DOAC Group: patients receiving uninterrupted DOAC therapy (apixaban, rivaroxaban, edoxaban, or dabigatran)
– Control Group: patients not receiving any anticoagulant therapy

Inclusion criteria were age ≥ 18 years; capacity to understand and sign the informed consent form; documented indication for oral soft tissue biopsy. Exclusion criteria were inability to provide consent; congenital or acquired coagulopathy (including haemophilia, von Willebrand disease, thrombocytopenia, or hepatic cirrhosis); active antiplatelet therapy other than low‐dose aspirin. DOAC therapy was not modified in the perioperative period.

### 2.3 Pre‐Operative Assessment

All patients underwent standardised pre‐operative assessment including a comprehensive medical history questionnaire, oral clinical examination and routine blood testing including complete blood count with differential, coagulation profile (prothrombin time [PT], activated partial thromboplastin time [aPTT], fibrinogen, INR), and serum creatinine. Pre‐operative parameters of both groups were recorded and compared.

### 2.4 Surgical Procedure

All biopsies were performed under local infiltrative anaesthesia with mepivacaine and vasoconstrictor administered circumferentially around the biopsy site. A standardised excisional technique was used: cold‐blade incision of the biopsy area, excision with atraumatic forceps, placement of the specimen in 10% formalin for histopathological processing, irrigation of the surgical site with sterile 0.9% saline, and closure with 4–0 resorbable Vicryl sutures (Figure 1). No haemostatic adjuncts (e.g., collagen sponge, oxidised cellulose) were applied prophylactically; tranexamic acid‐soaked gauze was reserved for cases of active perioperative bleeding. All surgeries were performed by the same oral surgeon with more than 10 years of experience (M.N.). All study parameters (except for intraoperative ones) were evaluated by two investigators who were not involved in the surgical procedures (R.I. and C.C.).

**FIGURE 1:**
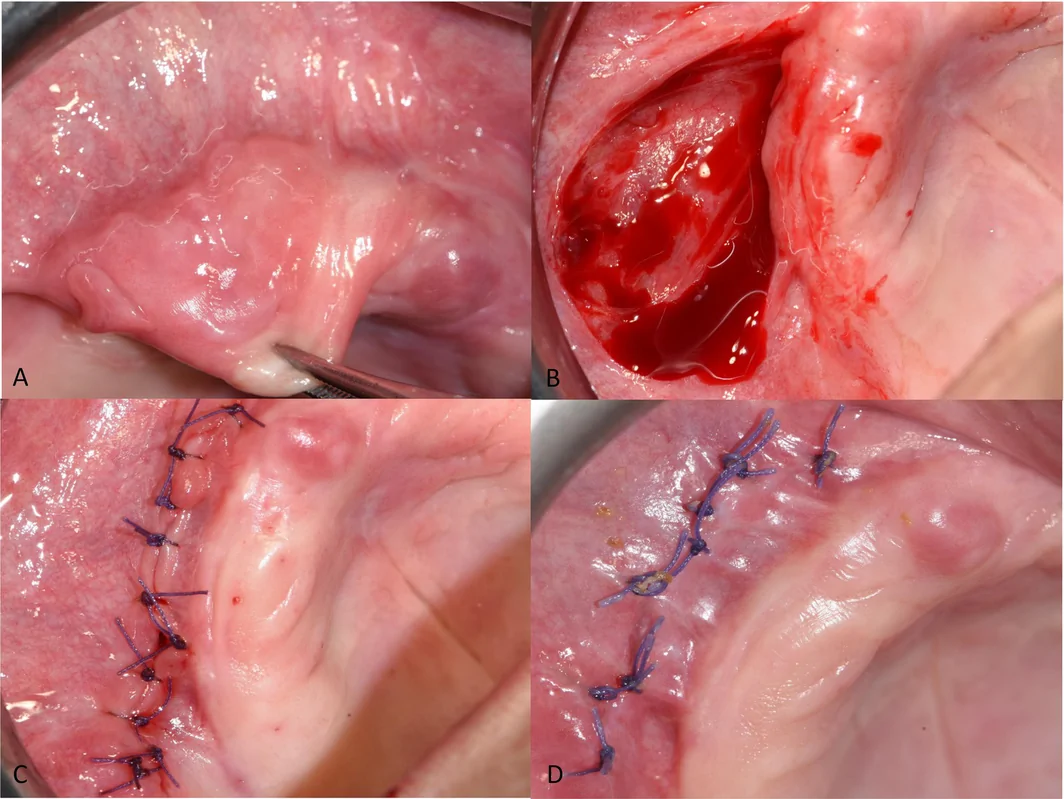
Clinical images of a soft tissue biopsy in a patient on DOAC therapy. (A) Initial presentation; (B) flap elevation and lesion exposure; (C) suturing; (D) 1‐week follow‐up.

### 2.5 Bleeding Assessment

Bleeding outcomes were categorised as follows: based on validated definitions adapted from previous dental surgery literature (Mauprivez et al. 2016; Cocero et al. 2019):

– Intraoperative bleeding: bleeding during the surgical procedure causing inadequate visualisation of the operative field and requiring active haemostatic intervention (gauze compression, diathermocoagulation, or haemostatic agents) (Figure 2).
– Perioperative bleeding (immediate postoperative): persistent oozing or frank bleeding within 80 min of procedure completion that could not be controlled by standard gauze compression alone, assessed dichotomously (present/absent) at 20‐, 40‐, 60‐ and 80‐min post‐procedure. When bleeding was detected at 20 min, tranexamic acid‐soaked gauze was applied; if bleeding persisted at 40 min, resuturing or diathermocoagulation was performed.
– Postoperative bleeding: any bleeding beyond 8–12 h from procedure completion (Kumbargere Nagraj et al. 2018), recorded daily by patients in a clinical diary from day 1 (T1) to day 7 (T7) and graded as follows: absent; mild (manageable with gauze/tranexamic acid at home); moderate (exuberant clot formation or bleeding requiring outpatient review; see Figure 3). No severe bleeding (requiring hospitalisation, blood transfusion, or systemic reversal agents) was defined a priori.

**FIGURE 2:**
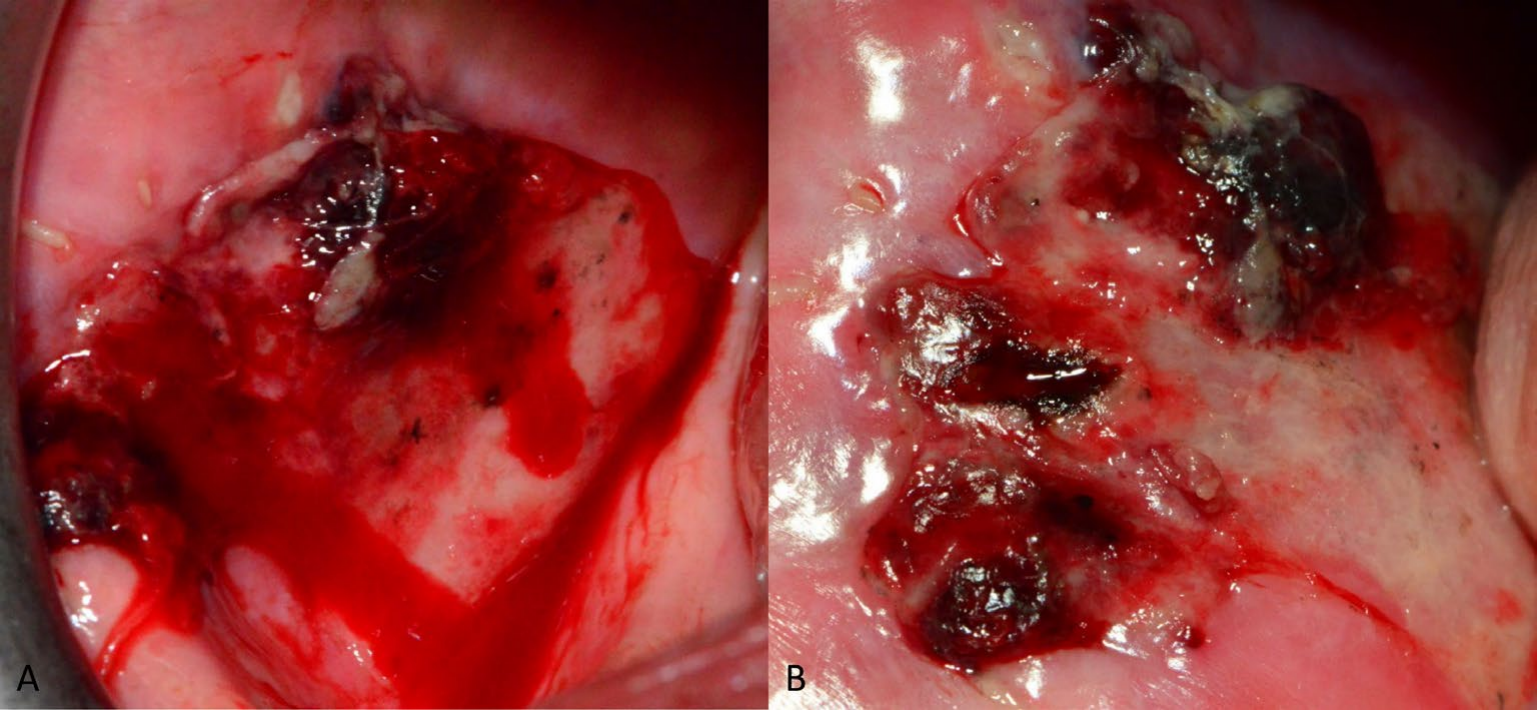
Management of intraoperative bleeding. (A) Haemorrhage following lesion removal from the buccal mucosa; (B) haemostasis achieved after diathermocoagulation.

**FIGURE 3:**
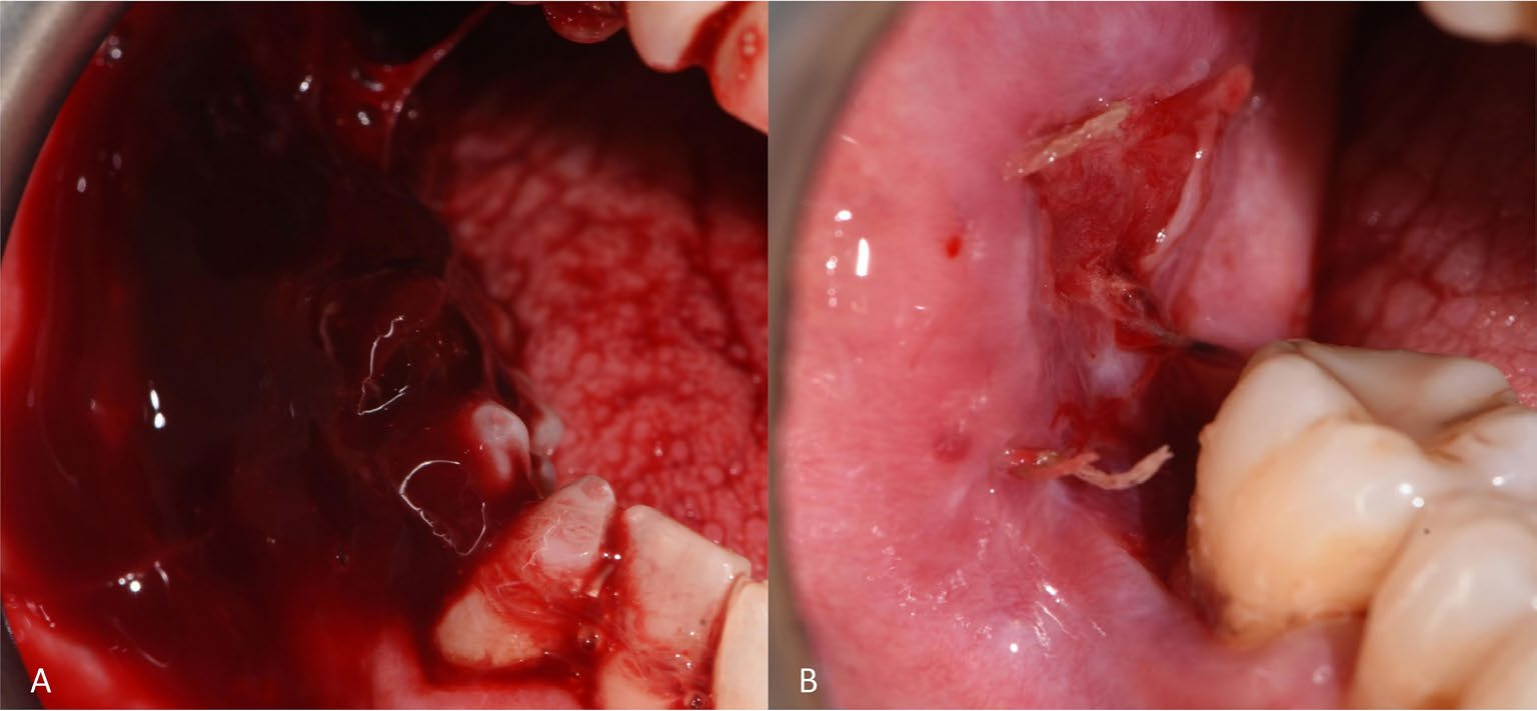
Management of moderate postoperative bleeding. (A) Haemorrhage and exuberant clot following lesion removal; (B) haemostasis achieved after coagulum removal and resuturing.

### 2.6 Sample Size Estimation

The sample size was determined a priori to detect a clinically significant difference in the primary outcome of perioperative bleeding at 20 min post‐procedure. Based on a review of the limited available literature on oral biopsies in anticoagulated patients and pilot data from our centre, we estimated a perioperative bleeding rate of 25% in the DOAC group versus 5% in the control group. These estimates were consistent with previously reported bleeding rates for minor oral surgery procedures in patients on uninterrupted anticoagulation (Mauprivez et al. 2016; López‐Galindo and Grau‐Benítez 2023).

Using a two‐sided test for independent proportions with a significance level (α) of 0.05 and a power (1‐β) of 0.80, the required sample size was calculated to be 46 patients per group. Accounting for a potential 10% dropout or incomplete data rate, the target enrolment was set at 50 patients per group.

### 2.7 Statistical Analysis

Data were entered into an Excel 2007 database (Microsoft, Redmond, WA) and imported into XLSTAT for Mac (Addinsoft, Paris, France; version 2021). Normality of distribution was assessed with the Shapiro–Wilk test. Between‐group comparisons for continuous variables were performed using the Kruskal‐Wallis test (non‐parametric). Given the prospective cohort design with independent (unpaired) groups, between‐group comparisons for categorical outcomes (including all bleeding endpoints) were performed using Chi‐square or Fisher's exact test as appropriate. Postoperative bleeding proportions from T1 to T7 were analysed as repeated binary observations; a Bonferroni correction was applied to account for multiple comparisons across time points. Statistical significance was set at *p* < 0.05.

## 3 Results

### 3.1 Demographic and Clinical Characteristics

One hundred patients completed the study (57 females, 43 males). The mean age was 74.6 years (SD 10.9; range 45–90) in the DOAC group and 72.2 years (SD 7.5; range 53–88) in the control group. No statistically significant differences were found between groups in terms of age (*p* = 0.129), sex distribution, or BMI (24.3 vs. 23.7 kg/m^2^, respectively). Demographic characteristics are summarised in Table 1.

**TABLE 1:** Demographic and clinical characteristics of the study population.

Variable	DOAC group (*n* = 50)	Control group (*n* = 50)	*p*
Age, years—mean ± SD (range)	74.6 ± 10.9 (45–90)	72.2 ± 7.5 (53–88)	0.129
Female, *n* (%)	27 (54%)	30 (60%)	0.672
BMI, kg/m^2^ – mean ± SD	24.3 ± 4.0	23.7 ± 3.5	0.366
Smoking, *n* (%)	6 (12%)	8 (16%)	0.775
Hypertension, *n* (%)	24 (48%)	18 (36%)	0.234
Diabetes mellitus, *n* (%)	11 (22%)	8 (16%)	0.597
Concomitant aspirin, *n* (%)	2 (4%)	0 (0%)	0.494
Apixaban/Rivaroxaban/Edoxaban/Dabigatran	17/14/10/9	N/A	—
DOAC duration, months – mean ± SD	34.7 ± 18.8	N/A	—

*Note: p*‐values: Kruskal‐Wallis for continuous, Fisher exact for categorical variables.

Abbreviations: BMI: body mass index; DOAC: direct oral anticoagulant; SD: standard deviation.

In the DOAC group, the most common comorbidities were hypertension (48%), diabetes mellitus (22%), and osteoporosis (14%). The principal indication for anticoagulant therapy was non‐valvular atrial fibrillation (NVAF) in 43 patients (86%) and VTE in 7 (14%). Drug distribution was: apixaban 34% (*n* = 17), rivaroxaban 28% (*n* = 14), edoxaban 20% (*n* = 10), and dabigatran 18% (*n* = 9); the vast majority received standard dosing. Mean duration of DOAC therapy was 34.7 months (SD 18.8). Two patients (4%) in the DOAC group were concomitantly receiving low‐dose aspirin. No patient in the control group was receiving anticoagulant or antiplatelet therapy.

### 3.2 Preoperative Laboratory Parameters

Preoperative haematological parameters are reported in Table 2. Both groups showed comparable values for red blood cell count, haemoglobin, platelet count, PT activity, fibrinogen and creatinine. Statistically significant differences were observed for INR (1.17 ± 0.11 vs. 1.07 ± 0.10, *p* < 0.001), aPTT in seconds (39.1 ± 13.6 vs. 28.6 ± 6.8, *p* < 0.001), and aPTT ratio (1.41 ± 0.48 vs. 1.06 ± 0.13, *p* < 0.001) in favour of higher values in the DOAC group, consistent with the anticoagulant mechanism of these agents.

**TABLE 2:** Preoperative laboratory parameters.

Parameter (mean ± SD)	DOAC group (*n* = 50)	Control group (*n* = 50)	*p*
Red blood cells (10^6^/μL)	4.53 ± 0.52	4.55 ± 0.37	0.398
Haemoglobin (g/dL)	13.8 ± 1.6	13.5 ± 1.5	0.144
Platelets (10^3^/μL)	265.6 ± 92.7	281.4 ± 83.5	0.211
PT activity (%)	97.1 ± 12.4	98.4 ± 9.5	0.312
PT ratio	1.10 ± 0.10	1.10 ± 0.07	0.130
INR	1.17 ± 0.11	1.07 ± 0.10	< 0.001*
aPTT (seconds)	39.1 ± 13.6	28.6 ± 6.8	< 0.001*
aPTT ratio	1.41 ± 0.48	1.06 ± 0.13	< 0.001*
Fibrinogen (mg/dL)	321.2 ± 81.7	329.7 ± 78.7	0.291
Creatinine (mg/dL)	0.94 ± 0.25	0.89 ± 0.21	0.150

Abbreviations: aPTT: activated partial thromboplastin time; INR: international normalised ratio; PT: prothrombin time; SD: standard deviation.

* *p* < 0.05 (Kruskal‐Wallis test), statistically significant.

### 3.3 Biopsy Site and Histopathological Diagnosis

The most common biopsy sites in the DOAC group were buccal mucosa (*n* = 18, 36%), gingiva (*n* = 13, 26%), and tongue (*n* = 9, 18%). In the control group, gingiva was the predominant site (*n* = 22, 44%), followed by buccal mucosa (*n* = 12, 24%) and tongue (*n* = 7, 14%). A statistically significant between‐group difference was found for gingiva (*p* < 0.001) and buccal mucosa (*p* < 0.05). The histopathological diagnoses varied widely; the most frequent diagnoses in the DOAC group were fibroma (*n* = 13), oral lichen planus (*n* = 10), and leucoplakia (*n* = 5). In the control group, oral lichen planus (*n* = 12), fibroma (*n* = 6) and pyogenic granuloma (*n* = 7) were most common. Statistically significant intergroup differences were observed for fibroma, pyogenic granuloma and vascular malformation (*p* < 0.05 for each).

### 3.4 Intraoperative and Perioperative Bleeding

Intraoperative and perioperative bleeding data are presented in Table 3. Intraoperative bleeding occurred in 8 DOAC patients (16%) compared to 3 controls (6%), a difference reaching statistical significance (*p* < 0.05). At 20 min post‐procedure, bleeding was detected in 12 DOAC patients (24%) versus 3 controls (6%, *p* < 0.05). The disparity widened progressively: at 40 min, 10 DOAC patients (20%) vs. 2 controls (4%, *p* < 0.001); at 60 min, 13 DOAC patients (26%) vs. 1 control (2%, *p* < 0.05); at 80 min, 12 DOAC patients (24%) vs. no control patients (0%, *p* < 0.05). In the DOAC group, haemostasis could not be achieved within 80 min in a subset of patients, necessitating prolonged monitoring and, in some cases, resuturing or diathermocoagulation.

**TABLE 3:** Intraoperative and perioperative bleeding outcomes.

Bleeding Assessment	DOAC group *n* (%)	Control group *n* (%)	*p*
Intraoperative	8 (16%)	3 (6%)	< 0.05*
Perioperative at 20 min	12 (24%)	3 (6%)	< 0.05*
Perioperative at 40 min	10 (20%)	2 (4%)	< 0.001*
Perioperative at 60 min	13 (26%)	1 (2%)	< 0.05*
Perioperative at 80 min	12 (24%)	0 (0%)	< 0.05*

* Statistically significant (*p* < 0.05). Fisher's exact test/McNemar's test.

### 3.5 Postoperative Bleeding

Postoperative bleeding data are summarised in Table 4. In the DOAC group, 22% of patients experienced bleeding on day 1 (T1) compared to 6% of controls (*p* < 0.05). At T2, 16% of DOAC patients reported bleeding vs. 4% of controls (*p* < 0.05). At T3 and T4, differences did not reach conventional significance (*p* = 0.281 and *p* = 0.078, respectively), though numerically higher rates were observed in the DOAC group. Two DOAC patients (4%) experienced moderate postoperative bleeding—characterised by exuberant clot formation at T3 and T4—that required outpatient review, local haemostatic intervention, and repeat suturing; no corresponding events occurred in controls (*p* < 0.05). No patient in either group experienced severe bleeding requiring hospitalisation or DOAC reversal therapy. All bleeding events were ultimately resolved with local haemostatic measures.

**TABLE 4:** Postoperative bleeding outcomes (days 1–7).

Postoperative day	DOAC group *n* (%)	Control group *n* (%)	*p*
T1 (Day 1)	11 (22%)	3 (6%)	< 0.05*
T2 (Day 2)	8 (16%)	2 (4%)	< 0.05*
T3 (Day 3)	2 (4%)	1 (2%)	0.281
T4 (Day 4)	2 (4%)	0 (0%)	0.078
T5 (Day 5)	1 (2%)	0 (0%)	0.095
T6 (Day 6)	3 (6%)	0 (0%)	0.097
T7 (Day 7)	3 (6%)	0 (0%)	0.097
Moderate bleeding (exuberant clot) at T3–T4	2 (4%)	0 (0%)	< 0.05*

* Statistically significant (*p* < 0.05). McNemar's test for paired data. All moderate bleeding events managed with local haemostatic measures; no hospitalisation required.

## 4 Discussion

To our knowledge, this is the first prospective cohort study specifically designed to evaluate bleeding outcomes after oral soft tissue biopsy in patients on uninterrupted DOAC therapy. Our results demonstrate that DOAC use is associated with significantly higher rates of intraoperative, perioperative and early postoperative bleeding compared to anticoagulant‐naive controls, while confirming that these bleeding events remain mild to moderate in severity and are manageable with local haemostatic measures in an outpatient setting.

The clinical relevance of these findings must be contextualised. Recently, a shift towards DOAC prescribing has been observed: over 80% of newly anticoagulated patients now receive DOACs rather than VKAs (Mega and Simon 2015). Given the epidemiological association between conditions requiring anticoagulation (particularly NVAF) and older age, this clinical scenario will be increasingly encountered (Inchingolo et al. 2024).

It should be noted that both biopsy site and lesion type may independently influence bleeding risk. The two groups differed significantly in biopsy site distribution (gingiva, *p* < 0.001; buccal mucosa, *p* < 0.05) and histopathological diagnosis (fibroma, pyogenic granuloma and vascular malformations, *p* < 0.05 for each). The absence of formal group matching for these variables represents a limitation of the present study, and the observed intergroup differences in bleeding outcomes should be interpreted accordingly. However, it is worth noting that pyogenic granulomas and vascular malformations occurred exclusively or predominantly in the control group, which would be expected to increase—rather than decrease—the bleeding risk in controls. The fact that the DOAC group still demonstrated significantly higher bleeding rates despite this potential confounding in the opposite direction lends additional robustness to the main finding. Future prospective studies should implement formal matching or multivariate adjustment for lesion type and biopsy site.

The significantly elevated aPTT and INR values in our DOAC group, despite being within clinically acceptable ranges for DOAC‐treated patients, reflect the pharmacodynamic effect of these agents on coagulation cascade markers. DOACs, particularly direct thrombin inhibitors such as dabigatran and factor Xa inhibitors such as rivaroxaban, prolong aPTT and, to a lesser extent, INR (Ballestri et al. 2023). These laboratory alterations did not predict individual bleeding risk, but at a group level, they correlated with the observed differences in bleeding outcomes.

Our finding that intraoperative bleeding was significantly more frequent in DOAC patients (16% vs. 6%) is consistent with the known pharmacology of these agents. Factor Xa and thrombin inhibition impairs both the coagulation cascade and thrombin‐mediated platelet activation, producing a clinically relevant, if transient, haemostatic deficit at surgical wound sites (Ballestri et al. 2023). The persistently elevated perioperative bleeding rates from 20 to 80 min post‐procedure in the DOAC group (24% at 20 min, 20% at 40 min, 26% at 60 min and 24% at 80 min) are particularly noteworthy: the transient decrease at 40 min likely reflects the effect of initial haemostatic measures, while the subsequent rise at 60 min indicates that these measures may not be durable in all DOAC patients. This pattern suggests that extended post‐procedure monitoring—to at least 80 min, as per our protocol—is clinically prudent.

The postoperative bleeding data reveal a significant difference at T1 and T2, which corresponds to the period when tissue‐level fibrinolysis in oral mucosa is most active and before adequate healing has occurred. The peak at T1 (22% in DOAC vs. 6% in controls) is consistent with previous studies in dental extractions (Mauprivez et al. 2016; López‐Galindo and Grau‐Benítez 2023), where postoperative day 1 represents the highest‐risk window for secondary haemorrhage in anticoagulated patients. The two cases of moderate postoperative bleeding—characterised by exuberant clot formation at T3 and T4, which required outpatient review and local intervention—highlight that a minority of DOAC patients may experience delayed, more significant haemostatic failure. These events are consistent with a ‘moderate’ bleeding not requiring drug reversal or hospitalisation (Cocero et al. 2019).

The absence of severe bleeding in our cohort is an important and clinically reassuring finding. Although higher bleeding rates were observed in the DOACs group, the absolute increase in risk was manageable and confined to the mild‐to‐moderate spectrum. This parallels the experience reported in dental extraction studies, confirming that patients on DOACs have an increased relative risk of bleeding compared to non‐anticoagulated controls, but severe events requiring hospitalisation are rare (Izzetti et al. 2022, 2024). Our data extend this observation to the specific context of soft tissue biopsy.

The role of local haemostatic measures deserves emphasis (Boccatonda et al. 2023). Our protocol employed tranexamic acid‐soaked gauze as a first‐line response to perioperative bleeding, and resuturing or diathermocoagulation when bleeding persisted. Tranexamic acid, an antifibrinolytic agent that inhibits plasminogen activation, has been shown to be the most effective topical haemostatic agent in anticoagulated dental surgery patients in a network meta‐analysis (Mahardawi et al. 2023), and its use has been supported by multiple systematic reviews (de Vasconcellos et al. 2017; Statman 2023). The absence of any severe bleeding event in our cohort may in part reflect the efficacy of this structured local haemostatic protocol.

Current guidelines do not specifically address oral soft tissue biopsy. The 2021 EHRA Practical Guide on DOACs (Steffel et al. 2021) addresses perioperative management according to procedure type and bleeding risk category, and places routine dental procedures (extractions, scaling, implant placement) in the ‘minor risk’ category requiring no drug interruption. Our data suggest that soft tissue biopsies occupy a bleeding risk category intermediate between simple extractions (where continuation is clearly safe) and complex surgical procedures (where suspension is often warranted). Given that all our bleeding events were manageable without DOAC interruption, a practical recommendation based on our results would be to perform oral biopsies without DOAC suspension, provided that an extended perioperative monitoring protocol with tranexamic acid‐based haemostasis is in place, and that patients are clearly instructed on recognition and management of early postoperative bleeding.

Indeed, the recommendation for 80‐min post‐procedural monitoring may pose logistical and resource constraints in high‐volume outpatient settings. A pragmatic alternative for settings where 80‐min monitoring is not feasible would be to discharge patients at 40–60 min if haemostasis has been confirmed at the 40‐min check, with explicit written and verbal instructions on how to manage postoperative bleeding at home, including provision of pre‐soaked tranexamic acid gauze and clear escalation criteria for outpatient review. The cost‐effectiveness of the 80‐min protocol versus a structured early‐discharge approach with home haemostatic resources has not been formally evaluated and represents a worthwhile area for future investigation.

The comparison with gastrointestinal endoscopic biopsy provides a useful parallel: Konishi and collaborators (Konishi et al. 2023a, 2023b) demonstrated in a large propensity‐score matched cohort that endoscopic biopsy without DOAC withdrawal did not significantly increase postprocedural haemorrhage rates, supporting the premise that biopsies—when performed with haemostatic awareness—can be safely conducted in anticoagulated patients. Our data support a similar conclusion for the oral cavity.

Several limitations of this study should be acknowledged. The sample size of 50 patients per group, while adequate for detecting intraoperative and early postoperative differences, may be insufficient to power analyses for rare outcomes such as moderate postoperative bleeding. The sample size calculation was based exclusively on the primary outcome of perioperative bleeding at 20 min; secondary outcomes, including moderate postoperative bleeding, were not powered a priori and should therefore be interpreted with caution. The absence of differentiation by DOAC type and specific dosage within the DOAC group precludes subgroup analysis by individual agent, which would be of pharmacological interest. The study was conducted at a single tertiary referral centre, which may affect the generalisability of results to other clinical settings. Patient‐reported postoperative outcomes via clinical diary, while standardised, are subject to reporting bias. Moreover, this study did not capture thrombotic events following DOAC continuation, so the thrombotic safety of uninterrupted therapy is not directly addressed, although no thrombotic complications were clinically recorded. The control group was not formally matched to the DOAC group for lesion type, biopsy site, or lesion vascularity, which represent potential confounders that could independently influence bleeding risk and should be considered when interpreting group comparisons. Lesion size and biopsy specimen dimensions were not systematically recorded, precluding size‐based stratification of bleeding risk, as proposed by some authors (Serrano‐Sánchez et al. 2017); future studies should document these variables. The timing of the last DOAC dose prior to surgery was not uniformly recorded, which limits interpretation of pharmacokinetic effects on intraoperative bleeding; patients were instructed to take their morning dose as usual, but individual variation in dose‐to‐incision intervals cannot be excluded. The intraoperative bleeding definition relied partly on the operating surgeon's judgement regarding ‘inadequate visualisation’; although a single experienced surgeon performed all procedures, reducing inter‐operator variability, subjective assessment cannot be entirely excluded. Finally, two patients in the DOAC group were concomitantly receiving low‐dose aspirin; although this number is small and unlikely to materially affect group‐level results, the additive anti‐haemostatic effect of dual antithrombotic therapy cannot be entirely discounted.

## 5 Conclusions

The present prospective cohort study demonstrates that oral soft tissue biopsy in patients receiving uninterrupted DOAC therapy is associated with a statistically significant and clinically meaningful increase in intraoperative, perioperative and early postoperative bleeding compared to anticoagulant‐naïve controls. However, all bleeding events were mild to moderate in severity, manageable with local haemostatic measures in an outpatient setting, and no patient required hospitalisation, blood transfusion, or DOAC reversal therapy.

These findings support the hypothesis that oral soft tissue biopsy may be safely performed without DOAC discontinuation, provided that: (i) an extended perioperative monitoring protocol (at least 80 min post‐procedure) is in place; (ii) local haemostatic measures—including tranexamic acid‐soaked gauze and suturing—are readily available; and (iii) patients receive clear postoperative instructions regarding recognition and management of early bleeding episodes. It should be acknowledged, however, that DOAC patients required substantially more haemostatic interventions—including tranexamic acid application, re‐suturing and diathermocoagulation—and prolonged post‐procedural monitoring compared to controls. These differences represent a clinically meaningful increase in procedural burden that should inform pre‐operative counselling and resource planning, even when bleeding severity remains within a manageable range. Larger multicentre randomised or prospective studies, with subgroup analyses by DOAC type, lesion characteristics and biopsy site, are necessary to establish robust evidence‐based guidelines for this common clinical scenario.

## Author Contributions

**Rossana Izzetti:** conceptualization, investigation, methodology, validation, data curation, writing – original draft, formal analysis. **Chiara Cinquini:** investigation, visualization, writing – review and editing, data curation, conceptualization, software. **Marco Nisi:** supervision, conceptualization, methodology, investigation, validation, writing – original draft, project administration. **Antonio Barone:** conceptualization, validation, visualization, writing – review and editing, supervision, methodology.

## Funding

The authors have nothing to report.

## Ethics Statement

The study was approved by the institutional IRB (Ethics committee North‐West Tuscany area) with approval number 17796/2020.

## Consent

Written informed consent was obtained from all patients to participate in the present study.

## Conflicts of Interest

The authors declare no conflicts of interest.

## Data Availability

The data that support the findings of this study are available on request from the corresponding author. The data are not publicly available due to privacy or ethical restrictions.

## References

[odi70453-bib-0001] Ballestri, S. , E. Romagnoli , D. Arioli , et al. 2023. “Risk and Management of Bleeding Complications With Direct Oral Anticoagulants in Patients With Atrial Fibrillation and Venous Thromboembolism: A Narrative Review.” Advances in Therapy 40: 41–66. 10.1007/s12325-022-02333-9 PMC9569921 36244055

[odi70453-bib-0002] Boccatonda, A. , A. Frisone , F. Lorusso , et al. 2023. “Perioperative Management of Antithrombotic Therapy in Patients Who Undergo Dental Procedures: A Systematic Review of the Literature and Network Meta‐Analysis.” International Journal of Environmental Research and Public Health 20: 5293. 10.3390/ijerph20075293 PMC10093975 37047909

[odi70453-bib-0003] Cocero, N. , M. Basso , S. Grosso , and S. Carossa . 2019. “Direct Oral Anticoagulants and Medical Comorbidities in Patients Needing Dental Extractions: Management of the Risk of Bleeding.” Journal of Oral and Maxillofacial Surgery 77: 463–470. 10.1016/j.joms.2018.09.024 30347201

[odi70453-bib-0004] Connolly, S. J. , M. D. Ezekowitz , S. Yusuf , et al. 2009. “Dabigatran Versus Warfarin in Patients With Atrial Fibrillation.” New England Journal of Medicine 361: 1139–1151. 10.1056/NEJMoa0905561 19717844

[odi70453-bib-0005] Darwish, G. 2023. “The Effect of Direct Oral Anticoagulant Therapy (DOACs) on Oral Surgical Procedures: A Systematic Review.” BMC Oral Health 23: 743. 10.1186/s12903-023-03427-8 PMC10566068 37821865

[odi70453-bib-0006] de Vasconcellos, S. J. d. A. , T. de Santana Santos , D. M. Reinheimer , A. L. Faria‐e‐Silva , M. d. F. B. de Melo , and P. R. S. Martins‐Filho . 2017. “Topical Application of Tranexamic Acid in Anticoagulated Patients Undergoing Minor Oral Surgery: A Systematic Review and Meta‐Analysis of Randomized Clinical Trials.” Journal of Cranio‐Maxillofacial Surgery 45: 20–26. 10.1016/j.jcms.2016.10.001 27840121

[odi70453-bib-0007] Douketis, J. D. , A. C. Spyropoulos , M. H. Murad , et al. 2022. “Perioperative Management of Antithrombotic Therapy.” Chest 162: e207–e243. 10.1016/j.chest.2022.07.025 35964704

[odi70453-bib-0008] Galletti, G. , F. Alfonsi , A. Raffaele , et al. 2020. “Implant Placement in Patients Under Treatment With Rivaroxaban: A Retrospective Clinical Study.” International Journal of Environmental Research and Public Health 17: 4607. 10.3390/ijerph17124607 PMC7345116 32604907

[odi70453-bib-0009] Granger, C. B. , J. H. Alexander , J. J. V. McMurray , et al. 2011. “Apixaban Versus Warfarin in Patients With Atrial Fibrillation.” New England Journal of Medicine 365: 981–992. 10.1056/NEJMoa1107039 21870978

[odi70453-bib-0010] Hindricks, G. , T. Potpara , N. Dagres , et al. 2021. “2020 ESC Guidelines for the Diagnosis and Management of Atrial Fibrillation Developed in Collaboration With the European Association for Cardio‐Thoracic Surgery (EACTS).” European Heart Journal 42: 373–498. 10.1093/eurheartj/ehaa612 32860505

[odi70453-bib-0011] Inchingolo, F. , A. M. Inchingolo , F. Piras , et al. 2024. “Management of Patients Receiving Anticoagulation Therapy in Dental Practice: A Systematic Review.” Health 12: 1537. 10.3390/healthcare12151537 PMC11311453 39120240

[odi70453-bib-0012] Iwata, E. , A. Tachibana , J. Kusumoto , et al. 2022. “Risk Factors Associated With Post‐Extraction Bleeding in Patients on Warfarin or Direct‐Acting Oral Anticoagulants: A Retrospective Cohort Study.” Oral and Maxillofacial Surgery 26: 641–648. 10.1007/s10006-022-01039-0 35043275

[odi70453-bib-0013] Izzetti, R. , C. Cinquini , M. Nisi , M. Mattiozzi , M. Marotta , and A. Barone . 2024. “Direct Oral Anticoagulants and Bleeding Management Following Tooth Extractions—A Prospective Cohort Study.” Dentistry Journal 12: 279. 10.3390/dj12090279 PMC11430983 39329845

[odi70453-bib-0014] Izzetti, R. , M. Nisi , S. Gennai , and F. Graziani . 2022. “Evaluating the Relationship Between Mandibular Third Molar and Mandibular Canal With Semiautomatic Segmentation: A Pilot Study on CBCT Datasets.” Applied Sciences 12: 502.

[odi70453-bib-0015] Kearon, C. , E. A. Akl , J. Ornelas , et al. 2016. “Antithrombotic Therapy for VTE Disease.” Chest 149: 315–352. 10.1016/j.chest.2015.11.026 26867832

[odi70453-bib-0016] Kim, M.‐J. , M.‐K. Kim , and S.‐H. Kang . 2024. “Post‐Extraction Bleeding in Patients on Direct Oral Anticoagulants.” Journal of the Korean Association of Oral and Maxillofacial Surgeons 50: 189–196. 10.5125/jkaoms.2024.50.4.189 PMC11372225 39211967

[odi70453-bib-0017] Konishi, T. , S. Ono , A. Okada , et al. 2023a. “Comparison of Bleeding Following Gastrointestinal Endoscopic Biopsy in Patients Treated With and Without Direct Oral Anticoagulants.” Endosc Int Open 11: E52–E59. 10.1055/a-1981-2946 PMC9839429 36644535

[odi70453-bib-0018] Konishi, T. , S. Ono , A. Okada , et al. 2023b. “Erratum: Comparison of Bleeding Following Gastrointestinal Endoscopic Biopsy in Patients Treated With and Without Direct Oral Anticoagulants.” Endosc Int Open 11: C2. 10.1055/a-2031-2897 PMC10108598 37077907

[odi70453-bib-0019] Kumbargere Nagraj S , E Prashanti , H Aggarwal , et al. (2018). Interventions for Treating Post‐Extraction Bleeding. Cochrane Database of Systematic Reviews 3, no. 3: CD011930. 10.1002/14651858.CD011930.pub3. PMC6494262 29502332

[odi70453-bib-0020] López‐Galindo, M. , and M. Grau‐Benítez . 2023. “Systematic Review on the Effects of the Discontinuation of the Anticoagulant Therapy and the Postoperative Bleeding, in Patients Under New Oral Anticoagulants After Dental Extraction.” Journal of Clinical and Experimental Dentistry 15: e338–e345. 10.4317/jced.60122 PMC10155941 37152496

[odi70453-bib-0021] Mahardawi, B. , S. Jiaranuchart , S. Arunjaroensuk , K. A. Tompkins , A. Somboonsavatdee , and A. Pimkhaokham . 2023. “The Effect of Different Hemostatic Agents Following Dental Extraction in Patients Under Oral Antithrombotic Therapy: A Network Meta‐Analysis.” Scientific Reports 13: 12519. 10.1038/s41598-023-39023-7 PMC10397210 37532770

[odi70453-bib-0022] Mauprivez, C. , R. H. Khonsari , O. Razouk , P. Goudot , P. Lesclous , and V. Descroix . 2016. “Management of Dental Extraction in Patients Undergoing Anticoagulant Oral Direct Treatment: A Pilot Study.” Oral Surgery, Oral Medicine, Oral Pathology, Oral Radiology 122: e146–e155. 10.1016/j.oooo.2016.06.003 27554378

[odi70453-bib-0023] Mega, J. L. , and T. Simon . 2015. “Pharmacology of Antithrombotic Drugs: An Assessment of Oral Antiplatelet and Anticoagulant Treatments.” Lancet 386: 281–291. 10.1016/S0140-6736(15)60243-4 25777662

[odi70453-bib-0024] Nathwani, S. , and C. Wanis . 2017. “Novel Oral Anticoagulants and Exodontia: The Evidence.” British Dental Journal 222: 623–628. 10.1038/sj.bdj.2017.364 28428567

[odi70453-bib-0025] Patel, M. R. , K. W. Mahaffey , J. Garg , et al. 2011. “Rivaroxaban Versus Warfarin in Nonvalvular Atrial Fibrillation.” New England Journal of Medicine 365: 883–891. 10.1056/NEJMoa1009638 21830957

[odi70453-bib-0026] Schnabel, R. B. , X. Yin , P. Gona , et al. 2015. “50 Year Trends in Atrial Fibrillation Prevalence, Incidence, Risk Factors, and Mortality in the Framingham Heart Study: A Cohort Study.” Lancet 386: 154–162. 10.1016/S0140-6736(14)61774-8 PMC4553037 25960110

[odi70453-bib-0027] Serrano‐Sánchez, V. , J. Ripollés‐de Ramón , L. Collado‐Yurrita , et al. 2017. “New Horizons in Anticoagulation: Direct Oral Anticoagulants and Their Implications in Oral Surgery.” Medicina Oral, Patología Oral y Cirugía Bucal 22: e601–e608. 10.4317/medoral.21862 PMC5694183 28809374

[odi70453-bib-0028] Spyropoulos, A. C. , and J. D. Douketis . 2012. “How I Treat Anticoagulated Patients Undergoing an Elective Procedure or Surgery.” Blood 120: 2954–2962. 10.1182/blood-2012-06-415943 22932800

[odi70453-bib-0029] Statman, B. J. 2023. “Perioperative Management of Oral Antithrombotics in Dentistry and Oral Surgery: Part 2.” Anesthesia Progress 70: 37–48. 10.2344/anpr-70-01-06 PMC10069535 36995961

[odi70453-bib-0030] Steffel, J. , R. Collins , M. Antz , et al. 2021. “2021 European Heart Rhythm Association Practical Guide on the Use of Non‐Vitamin K Antagonist Oral Anticoagulants in Patients With Atrial Fibrillation.” EP Europace 23: 1612–1676. 10.1093/europace/euab065 PMC11636576 33895845

[odi70453-bib-0031] Woolcombe, S. A. , R. E. Ball , and J. P. Patel . 2022. “Managing Direct Oral Anticoagulants in Accordance With the Scottish Dental Clinical Effectiveness Programme Guidance for Patients Undergoing Dentoalveolar Surgery.” British Dental Journal 232: 547–554. 10.1038/s41415-022-3999-y PMC9033580 35459831

[odi70453-bib-0032] Zou, L. , and L. Hua . 2023. “Risk of Bleeding With Dental Implant Surgery in Patients on Anticoagulant or Antiplatelet Drugs: A Systematic Review and Meta‐Analysis.” Acta Odontologica Scandinavica 81: 98–104. 10.1080/00016357.2022.2085324 35763663

